# ALOX12: A Novel Insight in Bevacizumab Response, Immunotherapy Effect, and Prognosis of Colorectal Cancer

**DOI:** 10.3389/fimmu.2022.910582

**Published:** 2022-06-27

**Authors:** Siyuan Weng, Zaoqu Liu, Hui Xu, Xiaoyong Ge, Yuqing Ren, Qin Dang, Long Liu, Jian Zhang, Peng Luo, Jianzhuang Ren, Xinwei Han

**Affiliations:** ^1^ Department of Interventional Radiology, The First Affiliated Hospital of Zhengzhou University, Zhengzhou, China; ^2^ Interventional Institute of Zhengzhou University, Zhengzhou, China; ^3^ Interventional Treatment and Clinical Research Center of Henan Province, Zhengzhou, China; ^4^ Department of Respiratory and Critical Care Medicine, The First Affiliated Hospital of Zhengzhou University, Zhengzhou, China; ^5^ Department of Colorectal Surgery, The First Affiliated Hospital of Zhengzhou University, Zhengzhou, China; ^6^ Department of Hepatobiliary and Pancreatic Surgery, The First Affiliated Hospital of Zhengzhou University, Zhengzhou, China; ^7^ Department of Oncology, Zhujiang Hospital, Southern Medical University, Guangzhou, China

**Keywords:** ALOX12, colorectal cancer, bevacizumab, prognosis, immunotherapy, multi-omics, biomarker

## Abstract

Colorectal cancer is a highly malignant cancer with poor prognosis and mortality rates. As the first biological agent approved for metastatic colorectal cancer (mCRC), bevacizumab was confirmed to exhibit good performance when combined with chemotherapy and immunotherapy. However, the efficacy of both bevacizumab and immunotherapy is highly heterogeneous across CRC patients with different stages. Thus, exploring a novel biomarker to comprehensively assess the prognosis and bevacizumab and immunotherapy response of CRC is of great significance. In our study, weighted gene co-expression network analysis (WGCNA) and the receiver operating characteristic (ROC) curves were employed to identify bevacizumab-related genes. After verification in four public cohorts and our internal cohort, ALOX12 was identified as a key gene related to bevacizumab response. Prognostic analysis and *in vitro* experiments further demonstrated that ALOX12 was closely associated with the prognosis, tumor proliferation, invasion, and metastasis. Multi-omics data analysis based on mutation and copy number variation (CNV) revealed that RYR3 drove the expression of ALOX12 and the deletion of 17p12 inhibited ALOX12 expression, respectively. Moreover, we interrogated the relationship between ALOX12 and immune cells and checkpoints. The results exhibited that high ALOX12 expression predicted a higher immune infiltration and better immunotherapy response, which was further validated in Tumor Immune Dysfunction and Exclusion (TIDE) and Subclass Mapping (SubMap) methods. Above all, our study provides a stable biomarker for clinical protocol optimization, prognostic assessment, precise treatment, and individualized treatment of CRC.

## Introduction

Colorectal cancer (CRC) is reported to be the third leading cause of tumor mortality around the world with more than 850,000 deaths and 1.85 million cases annually. Twenty percent of patients with newly diagnosed CRC already have metastases. More seriously, nearly a quarter of CRC patients develop metastases after the onset of local disease (1). With the development of multidisciplinary and comprehensive treatment options, the survival of CRC patients has been considerably prolonged, while the long-term survival rate of CRC, especially metastatic patients, is still unsatisfactory (2). Bevacizumab is an anti-VEGF monoclonal antibody targeting angiogenesis (3). As a standard-of-care therapy in metastatic colorectal cancer (mCRC) patients and the first biological agent approved for mCRC, bevacizumab combined with chemotherapy (leucovorin, irinotecan, and fluorouracil) was confirmed to illustrate surprising performance (1, 3). In addition, studies with bevacizumab in mCRC have also shown the dramatic benefits of combining bevacizumab with new chemotherapy regimens (capecitabine/oxaliplatin or fluorouracil/leucovorin) as the first-line treatment and with leucovorin/fluorouracil and oxaliplatin as the second-line treatment (4–7). Unfortunately, despite the dramatic benefits in mCRC treatment, the molecular mechanism of bevacizumab is still unclear. Recently, Quintanilha et al. have reported that rs3795897 (G>A) in *AGAP1* might be a potential predictor related to bevacizumab and patient survival (8). It is worth noting that no bevacizumab response biomarker for CRC was reported before (8, 9). Nevertheless, just as the authors said, this study displayed many limitations and a lack of further validation, which make the result questionable. Considering this, exploring a novel biomarker to predict the response of bevacizumab for CRC is still warranted.

In recent years, immunotherapy has exhibited a great sensation due to the dramatic benefits of solid cancer treatments (2, 10). Immune checkpoint inhibitor (ICI) can promote the immune system to recognize and suppress basic targets of tumor cells such as PD-1, CTLA-4, and PD-L1 (11). In CRC, ICI therapy was approved for the treatment of patients with advanced microsatellite instability (MSI) or DNA mismatch repair (dMMR) deficiency in 2017 (12). Apart from this, there are other classification tools to stratify patients, such as molecular subtypes, *PD-1*, *PD-L1*, *CTLA-4*, and tumor mutation burden (TMB) (2). However, these classification systems do not perfectly predict response to ICI therapy and only a small proportion of CRC patients can benefit from them (13). Given the enormous cost and serious adverse effects of immunotherapy, exploring new biomarkers for effective immunotherapy management in CRC is warranted.

Due to the poor prognosis and high mortality rate of CRC patients, considerable effort has been invested to develop markers for assessing the prognosis of CRC over the past decade. It has been reported that mutations in *KRAS*, *PIK3CA*, and *BRAF*, amplification of *HER2*, and the loss of *SMAD4* were significantly associated with the relapse of CRC (14). The consensus molecular subtype (CMS) classification was confirmed to be related to the clinical outcome of CRC, and CMS4 tumors had a frustrating recurrence and overall survival (OS) (15). In addition, MSI-H patients were reported to illustrate a significantly reduced risk of recurrence and death (16). Recently, Liu et al. have reported that the double hit of TTN and OBSCN demonstrated a better prognosis in CRC (17). However, these biomarkers possess limited clinical utility and only a moderate accuracy of prediction (18, 19).

In the present study, considering the fundamental role of bevacizumab in mCRC treatment, the weighted gene co-expression network analysis (WGCNA) algorithm and receiver operating characteristic (ROC) curves were employed to identify bevacizumab-related genes. Subsequent validations in our internal cohort and four independent cohorts demonstrated the robust and accurate ability of ALOX12 in bevacizumab response prediction. Univariate and multivariate Cox analyses and survival analysis revealed that the high expression of ALOX12 predicted worse overall survival (OS), progression-free survival (PFS), and relapse-free survival (RFS). Additionally, gene set enrichment analysis (GSEA) and gene set variation analysis (GSVA) algorithms were employed to explore the potential functions, and two potential driving targets of ALOX12 (*RYR3* and *17p12*) were further determined based on multi-omics data analysis. Besides, we also investigated the tumor mutation burden (TMB), immune landscape, immune subtype, immune checkpoint profile, and potential drug targets of ALOX12. In conclusion, our study provides a stable and powerful biomarker for CRC patients to predict the bevacizumab response, prognosis (OS, RFS, and PFS), immunotherapy effects, and potential therapeutic agents, which performed a dramatic significance in clinical therapeutic regimen optimization, prognostic risk assessment, precision treatment, and the individualized treatment regimen formulation of CRC.

## Materials and Methods

### Data Collection and Processing

**Figure 1:**
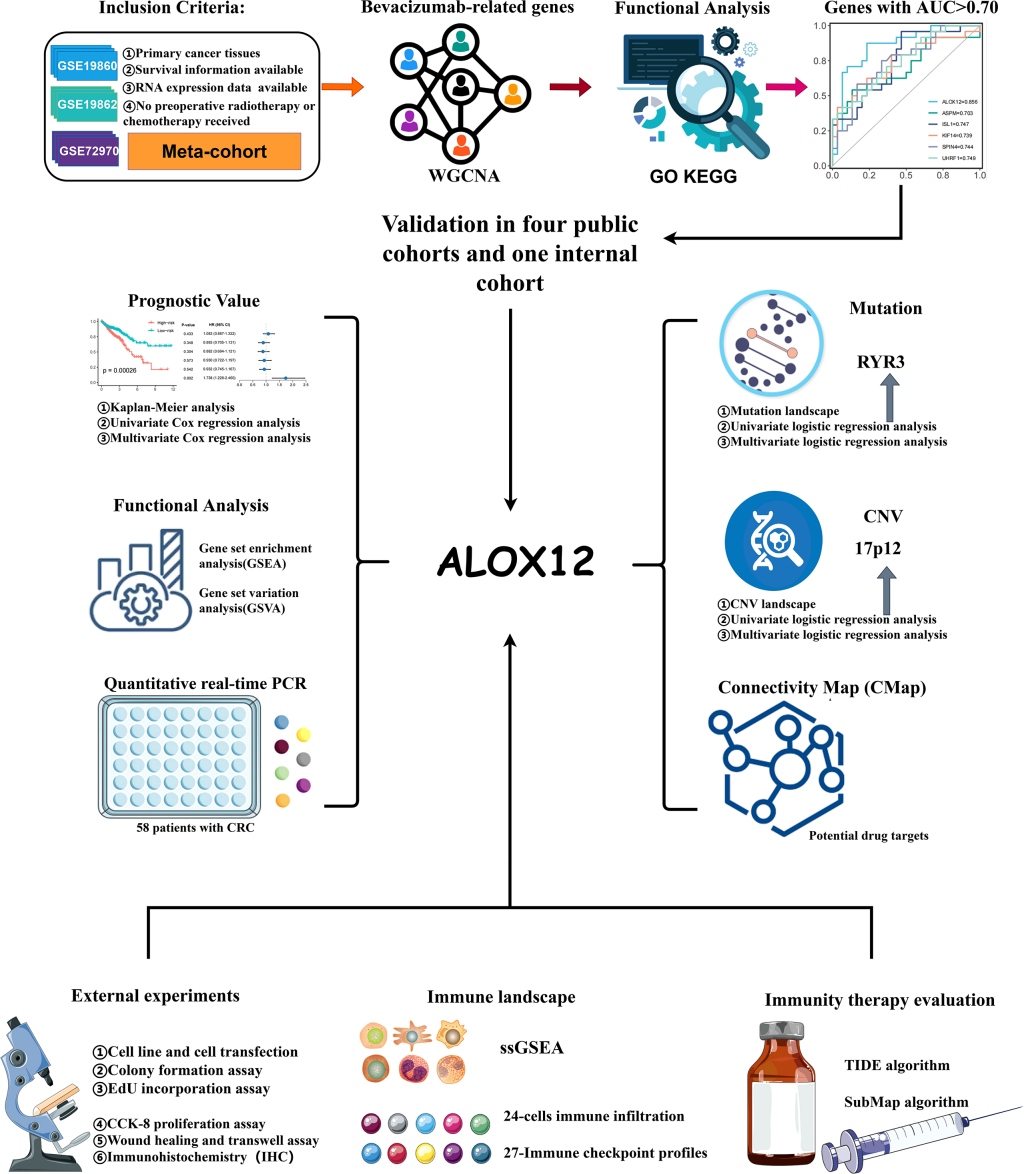
The flowchart of this study.

The flowchart of our study is illustrated in **Figure 1**. Three independent CRC cohorts were retrieved from the GEO website (http://www.ncbi.nlm.nih.gov/geo/), namely, GSE72970, GSE19860, and GSE19862. Two different CRC datasets were obtained from the UCSC Xena browser (https://xenabrowser.net/datapages/), namely, TCGA-COAD (n = 512) and TCGA-READ (n = 177). The somatic mutation (VarScan2 variant aggregation and masking) and HumanMethylation450 array were downloaded from TCGA GDC website (https://portal.gdc.cancer.gov/), and TMB was obtained by calculating the count of non-silent somatic mutation in every patient. Copy number variation (CNV) data processed by the Genomic Identification of Significant Targets in Cancer 2.0 (GISTIC2.0) algorithm were retrieved from FireBrowse (http://firebrowse.org/) (20). Of note, the robust multiarray averaging (RMA) algorithm implemented in the *affy* R package was utilized to process the raw data obtained from GEO, and the FPKM-normalized data from UCSC were further converted into log2 (TPM + 1).

Samples in TCGA cohort were screened according to the following conditions: (1) all samples were obtained from primary cancer tissues; (2) no preoperative radiotherapy or chemotherapy was received; (3) survival information was available; and (4) RNA expression data were available. Besides, in the GEO cohort, only samples with bevacizumab treatment response information were retained. For the detailed baseline data of all patients, please refer to **Table S1**.

### Construction of WGCNA

WGCNA is an R package available for weighted correlation network analysis. Before the construction of WGCNA, batch effects were removed from the meta-cohort (including GSE72970, GSE19860, and GSE19862) by the ComBat algorithm implemented in the *sva* package. In order to achieve the condition of a scale-free network, the optimal soft threshold β was identified and the adjacency matrix was transformed to a topological overlap matrix (TOM). Further, the corresponding dissimilarity (1-TOM) was calculated and modules were determined using the dynamic tree cutting method.

### Determination of Bevacizumab-Related Genes

After obtaining the modules, the relationship of the modules and bevacizumab treatment response was calculated. Afterward, three modules with the strongest correlation were selected and genes in these modules was defined as bevacizumab-related genes.

### Gene Ontology and Kyoto Encyclopedia of Genes and Genomes (KEGG) Enrichment Analysis

The molecular functions, biological processes, cellular components, and potential mechanism of bevacizumab-related genes were further explored by Gene Ontology (GO) and Kyoto Encyclopedia of Genes and Genomes (KEGG) analyses, which were conducted by the *clusterProfiler* package. Pathways with *P* < 0.05 were considered significant.

### ROC Curves, Cox Regression, and Survival Analysis

ROC curves and the area under the ROC curve (AUC) were employed to estimate the accuracy of bevacizumab-related genes for predicting bevacizumab treatment response, and genes with AUC >0.7 were retained, subsequently. After validating in GSE72970, GSE19860, GSE19862, meta-cohort, and our internal cohort, ALOX12 was identified as an accurate and stable predictor of bevacizumab response in CRC. Additionally, Kaplan–Meier survival analysis and univariate and multivariate Cox regression analyses were further employed to investigate the prognostic value of ALOX12.

### Gene Set Enrichment Analysis

Correlations between ALOX12 expression and all mRNA genes were evaluated by the Pearson correlation. All genes were arranged in descending order of correlation coefficient. Afterward, GSEA was conducted by the *clusterProfiler* R package to recognize remarkably enriched terms associated with the GO and KEGG pathways (21).

### Gene Set Variation Analysis

To investigate whether ALOX12 expression was associated with tumors, we performed GSVA *via* the *GSVA* R package (22). The hallmark gene set was obtained from the Molecular Signatures Database (MSigDB, https://www.gsea-msigdb.org/gsea/msigdb/index.jsp). Patients were divided into two groups according to the median expression of ALOX12. To reduce the overlap and redundancy of pathways, the gene set associated with a pathway was screened to contain only unique genes, and all genes related to multiple pathways were removed (23). The *limma* package was employed to recognize the remarkably altered pathways between the high and low groups, and the pathway with | t | >1 was regarded significant.

### The Mutation Landscape of CRC

The TMB of each patient was assessed using the *maftools* R package (24). To explore whether there were differences in genomic mutations between high and low ALXO12 expression groups, the mutation waterfall plot of the top 30 genes with the highest mutation number in CRC was visualized using the *maftools* and *ComplexHeatmap* packages. Subsequently, Wilcoxon test and univariate and multivariate logistic regression analyses were performed to assess and verify the correlation between 30-gene mutation status and ALOX12 expression, respectively. It is worth noting that apart from age, gender, and stage, TMB was also included in the multivariate logistic regression to ensure that the relationship between mutation and ALOX12 was independent.

### Copy Number Variation in CRC Patients

To investigate the proportion of genomic alterations in CRC, the fraction of genomic alterations (FGA), genomes gained (FGG), and genomes lost (FGL) were calculated, respectively. The *ComplexHeatmap* package was employed to visualize the CNV waterfall chart of the top 15 amplification (AMP) and homozygous deletion (Homdel) chromosome fragments in CRC. In addition, Wilcoxon test and univariate and multivariate logistic regression analyses were performed to calculate and confirm the correlation between the CNV of 30 fragments and ALOX12 expression. Of note, in addition to age, gender, and stage, FGG was contained in the multivariate logistic regression analysis to ensure that the correlation between ALOX12 expression and the AMP fragments was independent. Similarly, FGL was contained in the multivariate logistic regression analysis to ensure that ALOX12 was an independent factor of Homdel fragments.

### Comprehensive Analysis Based on Immune Infiltration and Immune Checkpoints

The single-sample gene set enrichment analysis (ssGSEA) algorithm was conducted to estimate the infiltration abundance of 24 immune cells in the tumor immune microenvironment *via* the *GSVA* package (25). The gene set of 24 immune cell types was obtained from the previous study (26). We also retrieved 27 immune checkpoints from the published studies, including the member of the B7-CD28 family (27), TNF superfamily (28), and other molecules (29, 30). Studies of the relationship between the ALOX12 expression and immune infiltration and checkpoints were employed subsequently.

### Immunotherapeutic Response Prediction

The tumor immune dysfunction and exclusion (TIDE) and subclass mapping (SubMap) algorithm were employed to predict the responses to ICB therapy (31, 32). Actually, TIDE evaluates immune evasion by integrating the expression characteristics of T-cell exclusion and T-cell dysfunction. In parallel, the GSEA algorithm was implemented in SubMap to derive the degree of commonality between high and low groups, and the adjusted *P-*value was employed to assess the similarity. A lower adjusted *P* value represents higher similarity.

### Connectivity Map Analysis Identified Potential Compounds/Inhibitors for CRC

Connectivity Map (CMap) is a public online tool that allows users to predict compounds that can activate or inhibit based on a gene expression signature (33). Based on the key gene expression, we performed CMap to screen potential therapeutic agents to further identify which target drug might be helpful against CRC. Agents with *P* < 0.001 were considered significant.

### Human Tissue Specimens and qRT−PCR Analysis

A total of 58 paired CRC tissues and matched adjacent non-tumor tissues were collected from the First Affiliated Hospital of Zhengzhou University. All patients signed informed consent. After radical surgery, patients received available standard systemic bevacizumab therapies. Drug responses were evaluated based on the Response Evaluation Criteria in Solid Tumors (RECIST, version 1.1). The detailed baseline characteristics of the patients are illustrated in **Table S1**. In the qRT-PCR analysis, six bevacizumab-related genes with AUC >0.7 were detected. Gene expression values were normalized to GAPDH and further log2 transformed. The primer sequences of the included six genes and GAPDH are exhibited in **Table S2**. See **Supplementary Material** for a detailed description.

### Immunohistochemistry

The anti‐ALOX12 (Ab211506, 1:100) antibody was employed to conduct immunohistochemistry (IHC). Percent staining was scored as follows: 1 (1%–25%), 2 (26%–50%), 3 (51%–75%), and 4 (76%–100%), and staining intensity was scored on a scale of 0 (signal-less color) to 3 (light yellow, brown, and dark brown). Stained tissue was scored by three individuals blinded to clinical parameters, and IHC scores were determined by percentage and intensity scores.

### Cell Lines and Cell Transfection

Two cell lines, namely, human CRC HCT-116 and SW480, were used in our research, which were cultured in RP1640 (Solarbio, Beijing, China, 31800-500) containing 10% FBS (04-001-1ACS, Bioind, Beit Haemek, Israel) at 37°C with 5% CO_2_. Silencer Select small interfering RNAs (siRNAs) specific for ENSG00000108839 (ALOX12) and inhibitor control were generated from RiboBio (Guangzhou, China). To silence mRNA in cancer cells, specific siRNA and control siRNA were transfected into HCT-116 and SW480 cells. Lipofectamine 3000 (Invitrogen, Carlsbad, USA) was utilized as a transfection carrier, and qRT-PCR analysis was employed to confirm the transfection efficiency.

### Wound Healing and Transwell Assay

Tumor cells were seeded in six-well plates, scraped with a sterile 200-μl pipette tip, and cultured in serum-free medium, and the wound width was measured at 0 and 48 h, respectively. The migration and invasive abilities of CRC cells were determined *via* transwell assays after transfection with siRNAs. See **Supplementary Material** for a detailed description.

### Colony Formation Assay

Equal numbers of transfected cells were inoculated into six-well plates at a density of 1,000 cells per well and incubated at 37°C for 2 weeks. Then, the cells were fixed with 4% paraformaldehyde for 15 min and stained with GIMSA for 10–30 min. Finally, the colonies were photographed and counted.

### Cell Counting Kit-8 Proliferation Assay and 5-Ethynyl-20-deoxyuridine Incorporation Assay

Cell proliferation was determined by Cell Counting Kit-8 (Fu Heng, Shanghai, China), and the Cell-Light EdU Apollo567 *In Vitro* Kit (RiboBio, Guangzhou, China) was used to assess the proliferation of cells according to the manufacturer’s instructions. For detailed steps, please refer to the **Supplementary Material**.

### Statistical Analysis

The correlations between two variables were evaluated by Pearson correlation. The *Survival* package was utilized to perform Kaplan–Meier survival analysis, and the different significance was determined by the log-rank test. The ROC curves were plotted by the *pROC* package. Besides, the AUC was utilized to compare the accuracy for predicting the bevacizumab response of hub genes. Differences in key gene expression between the high and low groups were compared by independent-sample T-test or Wilcoxon rank-sum test. Multiple comparisons were conducted using Kruskal–Wallis tests. All statistical *P* values were two-sided, and *P* < 0.05 was defined as statistically significant. Adjusted *P*-value was employed using the Benjamini-Hochberg (BH) multiple-test correction. All data processing and plotting were finished in R 4.0.5 software.

## Results

### Construction of Gene Co-Expression Modules

**Figure 2:**
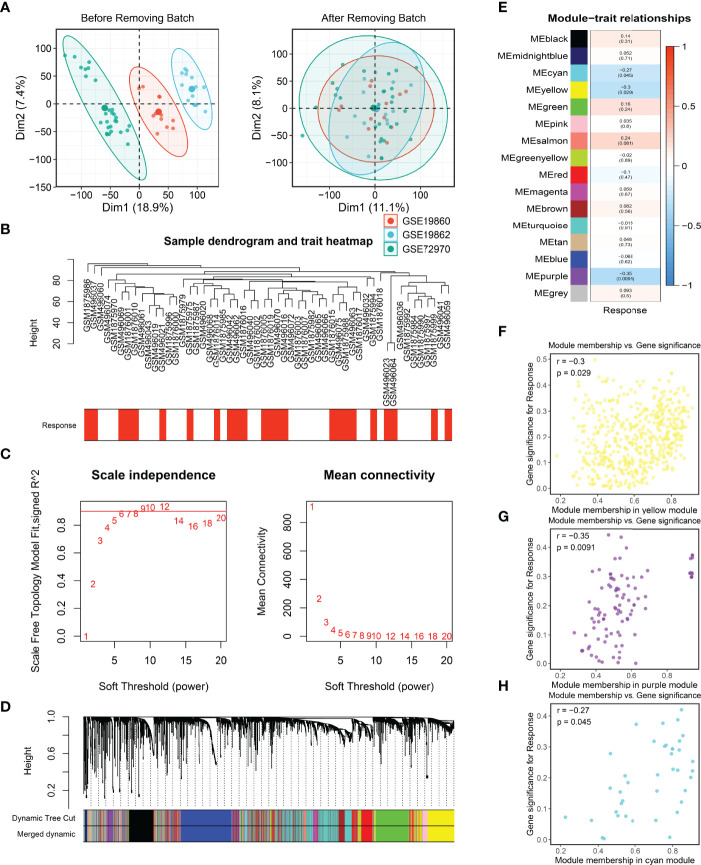
Batch correction and the identification of bevacizumab-related genes. **(A, B)** Batch removing of GSE19860, GSE19862, and GSE72970 **(A)**, and the sample dendrogram and trait heatmap of the meta-cohort **(B)**. **(C)** Scale-free topology criterion of the co-expression network, and an SFT in review plot for choosing the power β for the unsigned weighted correlation network. **(D)** Cluster dendrogram of genes in the co-expression network. **(E)** The correlation between modules and bevacizumab response in CRC. **(F–H)** Scatter plots of bevacizumab-related genes.

Firstly, three cohorts, namely, GES19860, GSE19862, and GSE72970, were combined into a meta-cohort and the batch effects were removed (**Figures 2A, B**). Afterward, the median absolute deviation (MAD) of the expression values for each gene was calculated, and the top 5,000 genes were taken for WGCNA after sorting in descending order. In the present study, a power of β = 6 was selected as the soft threshold to implement a scale-free network (**Figure 2C**) and the adjacency matrix was further transformed into a topological overlap matrix (**Figure S2A**). After calculating the signature genes of modules, the highly co-expressed genes were clustered into the same module by dynamic-tree cutting method (**Figure 2D**). Of note, the heatmap of the correlation for module traits showed that the purple, yellow, and cyan modules were tightly related to the response of bevacizumab in CRC (**Figure 2E**). The same results were also illustrated in the scatter plots of gene significance and module memberships (**Figures 2F–H**). Eventually, a total of 597 genes in purple, yellow, and cyan modules were defined as bevacizumab response-related genes.

### GO and KEGG Enrichment Analyses

**Figure 3:**
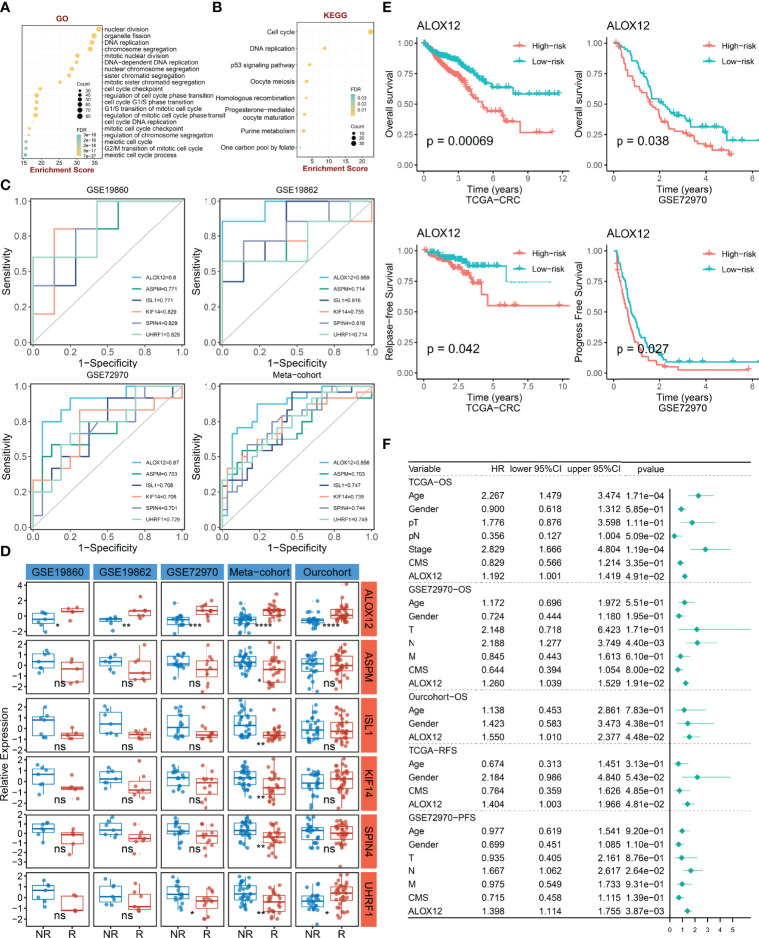
The identification and prognostic value assessment of ALXO12. **(A, B)** GO **(A)** and KEGG **(B)** enrichment analysis results of bevacizumab-related genes. **(C)** Bevacizumab-related genes with AUC >0.70. **(D)** Validation of the correlation between six-hub genes and bevacizumab response in four external cohorts and our internal cohort. *P < 0.05, **P < 0.01, ***P < 0.001, ****P < 0.0001. **(E)** Kaplan–Meier survival analysis of the high and low ALOX12 expression groups in TCGA-CRC and GSE72970. **(F)** Multivariate Cox regression analysis of OS, RFS, and PFS in TCGA-CRC, GSE72970, and our cohort. ns, no significance.

To explore the potential biological functions and potential molecular mechanisms of bevacizumab-related genes, GO and KEGG enrichment analyses were further employed. The result of GO analysis performed that bevacizumab-related genes were mainly enriched in DNA replication, chromosome segregation, cell-cycle checkpoint, and the regulation of cell-cycle phase transition pathways (**Figure 3A**). In parallel, the result of KEGG analysis exhibited that these genes were mostly enriched in the p53 signaling pathway, cell cycle, purine metabolism, and DNA replication pathways (**Figure 3B**). Taken together, GO and KEGG enrichment analyses indicated that bevacizumab-related genes might play an important role in the occurrence and development of tumors, as well as the proliferation and division of cancer cells.

### ALOX12, a Hub Gene Tightly Associated With Bevacizumab Response and Prognosis

To further verify the correlation between bevacizumab-related genes and bevacizumab response in CRC, ROC curves and AUC were plotted in four cohorts (including GSE19860, GSE19862, GSE72970, and meta-cohort). Under the screening conditions of AUC >0.7, six genes (namely, *ALOX12*, *ASPM*, *ISL1*, *KIF14*, *SPIN4*, and *UHRF1*) were retained (**Figure 3C**). Patients were divided into high and low groups according to the median expression of these six genes, respectively. As illustrated in **Figure 3D**, the differences of bevacizumab response in the two groups were dramatically significant in the four external cohorts and our internal cohort, which indicated that ALOX12 was an accurate and stable biomarker in predicting the response to bevacizumab for CRC. Of note, in our in-house cohort, ALOX12 expression was significantly elevated in CRC compared with normal tissues, and it was further validated in IHC (**Figure S1**). Additionally, we explored the prognostic value of ALOX12. Kaplan–Meier analysis suggested that a high expression of ALOX12 predicted worse OS, RFS, and PFS (**Figures 3E**, **S2B**). Univariate and multivariate Cox regression analyses suggested that ALOX12 was not only dramatically significant in predicting OS, RFS, and PFS (**Figures S2C–G**) but also an independent prognostic factor for CRC patients after adjusting other clinical characteristics (**Figure 3F**).

### Biological Function Analysis of ALOX12

**Figure 4:**
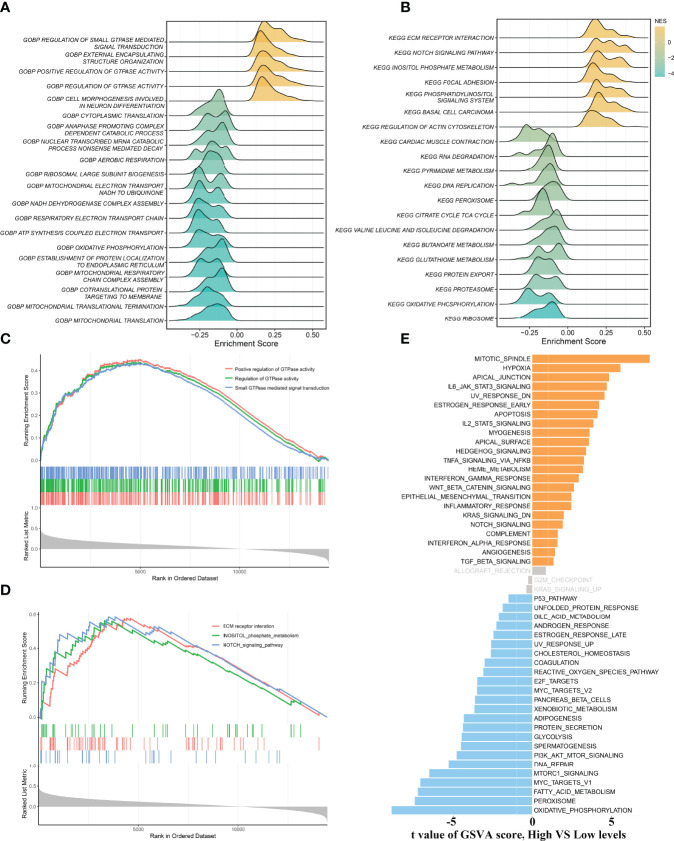
Gene set enrichment analysis (GSEA) and gene set variation analysis (GSVA). **(A, B)** The results of GO **(A)** and KEGG **(B)** enrichment analysis by the GSEA algorithm. **(C, D)** The significantly enriched pathways are associated with ALOX12 expression. **(E)** The result of GSVA between high and low ALOX12 expression groups.

To gain more insights into the potential functional characteristics and molecular mechanisms of ALOX12, GSEA and GSVA were employed, subsequently. As illustrated in **Figures 4A, B**, the top 20 pathways of GO and KEGG were selected separately according to the absolute value of normalized enrichment score (NES), from which we could observe that ALOX12 expression was tightly associated with tumor development, progression, metastasis, and immune-related pathways such as positive regulation of GTPase activity, small GTPase-mediated signal transduction, NOTCH signaling pathway, regulation of GTPase activity, ECM receptor interaction, and inositol phosphate metabolism pathways. As shown in **Figures 4C, D**, all these pathways were positively correlated with ALOX12 expression. Additionally, the GSVA result demonstrated that the vast majority of pathways in the hallmark gene set were significantly different between the high and low groups, indicating that ALOX12 expression was highly correlated with tumors (**Figure 4E**).

### ALOX12 Promoted the Proliferation, Invasion, and Metastasis of CRC

**Figure 5:**
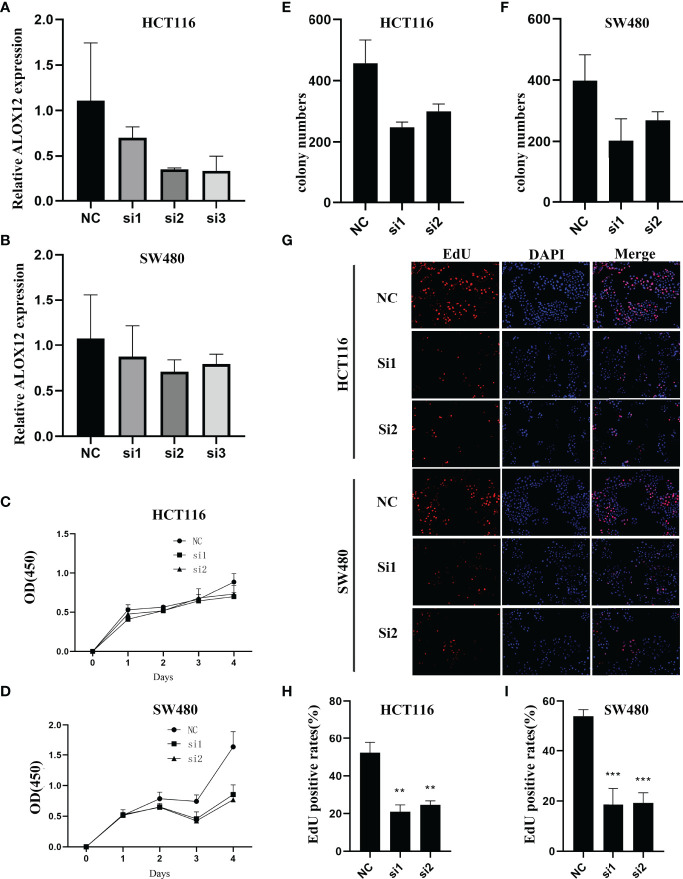
External experiment validation of the correlation between ALOX12 expression and tumor cell proliferation. **(A, B)** Cell transfection of HCT-116 **(A)** and SW480 **(B)** CRC cell lines. **(C, D)** CCK-8 proliferation assay of HCT-116 **(C)** and SW480 **(D)** cells. **(E)** Colony formation assay of HCT-116 **(E)** and SW480 **(F)** cells. **(G–I)** 5-Ethynyl-20-deoxyuridine (EdU) incorporation assay in HCT-116 and SW480 CRC cell lines. **P < 0.01, ***P < 0.001.

**Figure 6:**
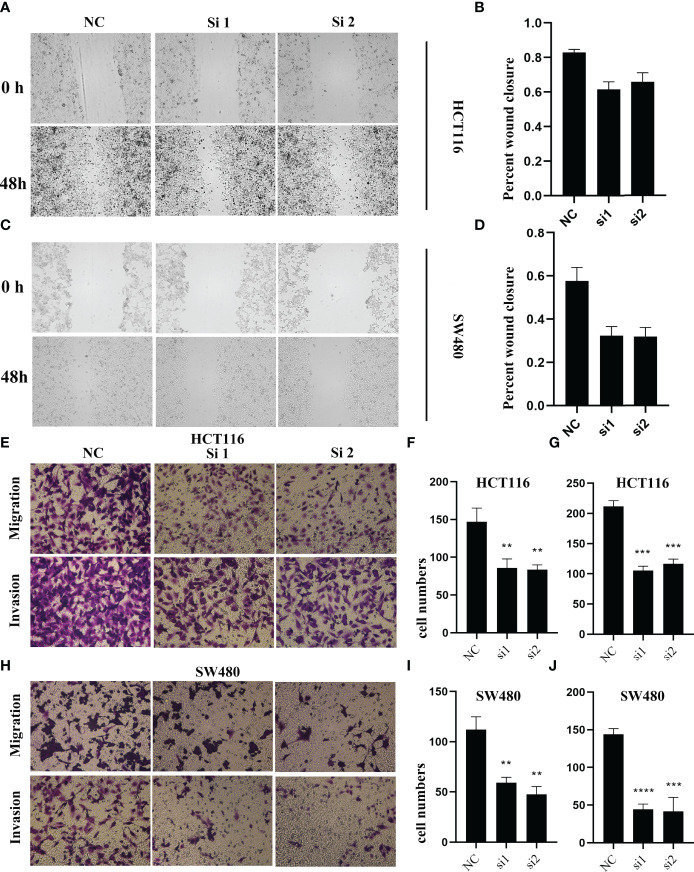
The result of wound healing **(A–D)** and transwell assay **(E–J)** in HCT-116 and SW480 CRC cell lines. **P < 0.01, ***P < 0.001, ****P < 0.0001.

To validate the biological function of ALOX12 in CRC cells, three special siRNAs were designed to knock down the expression of ALOX12 in HCT-116 and SW480 cell lines. As exhibited in **Figures 5A, B**, siRNA2 and siRNA3 efficiently reduced the ALOX12 expression in HCT-116 and SW480 cells. The growth curves of CCK8 assays demonstrated that down-regulation of ALOX12 suppressed the proliferation viability of HCT-116 and SW480 CRC cells (**Figures 5C, D**). Colony formation assays exhibited that the cell colony numbers of HCT-116 and SW480 cells were dramatically inhibited by the down-regulation of ALOX12 (**Figures 5E, F**). In parallel, 5-ethynyl-20-deoxyuridine (EdU) assays showed that knockdown of ALOX12 impaired the ratio of the positive cells (**Figures 5G–I**). Moreover, wound-healing assays suggested that knockdown of ALOX12 inhibited the migration of CRC cells (**Figures 6A–D**), and transwell assays, including migration and invasion assays, indicated that the migratory ability and invasive ability were reduced when ALOX12 was depleted (**Figures 6E–J**). Taken together, ALOX12 facilitated the proliferation, invasion, and metastasis of CRC.

### Somatic Mutational Landscape With Regard to ALOX12

**Figure 7:**
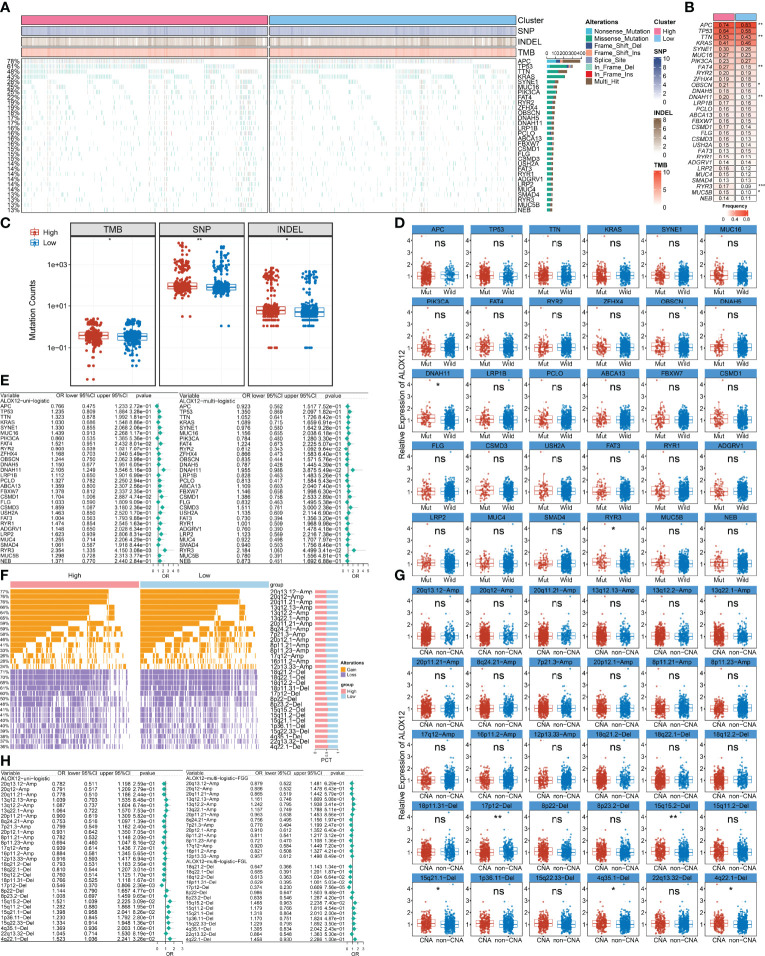
Identification of ALOX12-related mutation and copy number variation driver targets. **(A)** The mutational landscape of the top 30 frequently mutated genes (FMGs). **(B)** The mutation frequency of 30 FMGs between the high and low ALOX12 expression groups. **(C)** Boxplot of TMB, SNP, and INDEL between the high- and low-risk groups. **(D)** Identification of ALOX12 expression-related mutations. **(E)** Determination of independent ALOX12 expression-related gene mutation through univariate and multivariate logistic regression analyses. **(F)** The CNV landscape of the top 15 AMP and Homdel chromosome fragments between two ALOX12 expression groups. **(G)** Identification of ALOX12 expression-related CNV chromosome segments. **(H)** Determination of independent ALOX12 expression-related CNV chromosome segment through univariate and multivariate logistic regression analyses. *P < 0.05, **P < 0.01, ***P < 0.001. ns, no significance.

We further explored the mutational landscape of the top 30 frequently mutated genes (FMGs) (**Figure 7A**). Overall, seven FMGs exhibited a significantly higher mutational frequency between the high and low ALOX12 expression groups, including *APC*, *TTN*, *FAT4*, *OBSCN*, *DNAH11*, *RYR3*, and *MUC5B* (**Figure 7B**). Consistent with the mutation characteristics of these FMGs, the high-risk group also exhibited a generally superior burden including TMB (*P* < 0.05), single-nucleotide polymorphisms (SNPs, *P* < 0.01), and insertions and deletions (Indels, *P* < 0.05) (**Figure 7C**). Furthermore, we investigated the correlation between mutation status of the top 30 FMGs and ALOX12 expression. As illustrated in **Figure 7D**, *DNAH11* and *RYR3* were more likely to be mutated in CRC patients with high ALOX12 expression (*P* < 0.05). Besides, univariate and multivariate logistic regression revealed that a high expression of ALOX12 was not only tightly associated with RYR3 mutation but also remained an independent significance after adjusting for clinical characteristics such as age, gender, stage, and TMB (**Figure 7E**). Taken together, RYR3 mutation might drive the expression of ALOX12, and the higher ALOX12 expression predicted a superior mutational landscape.

### Deletion of 17p12 Inhibited the Expression of ALOX12

Furthermore, we characterized the CNV status of the top 15 AMP and Homdel chromosome fragments between two ALOX12 expression groups (**Figure 7F**). As illustrated in **Figure 7G**, deletion of 17p-12, 15q15.2, 15q21.1, 4q35.1, and 4q22.1 displayed significant differences between the high- and low-risk groups. Univariate and multivariate logistic regression suggested that a low expression of ALOX12 not only predicted the deletion of 17p12 but also remained an independent significance after adjusting for clinical characteristics such as age, stage, gender, and FGL (**Figure 7H**). Taken together, the deletion of 17p12 inhibited the expression of ALOX12.

### ALOX12 Expression Is Significantly Associated With Immune Infiltration

**Figure 8:**
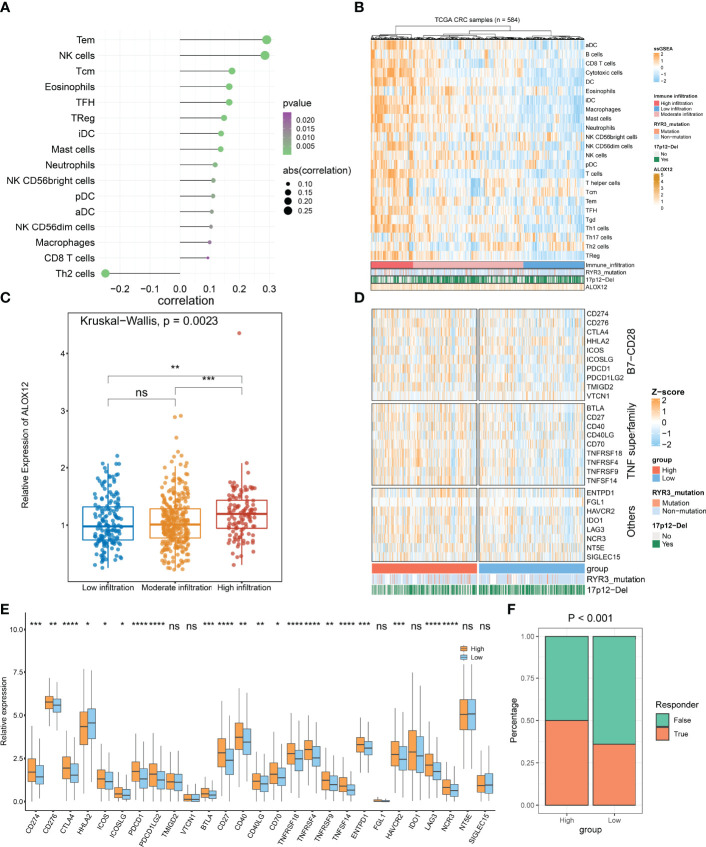
Tumor immune microenvironment landscape, immune checkpoint profiles, and immunotherapy response prediction of ALOX12 in TCGA-CRC. **(A–C)** The correlation analysis between ALOX12 and 24-immune-cell infiltration abundance. **(D, E)** Heatmap and boxplot of 27 immune checkpoint profiles in high and low ALOX12 expression groups. **(F)** The result of the tumor immune dysfunction and exclusion (TIDE) method. *P < 0.05, **P < 0.01, ***P < 0.001, ****P < 0.0001. ns, no significance..

On the basis of GSEA and GSVA results, we observed that ALOX12 expression was tightly associated with tumor immune-related pathways. Therefore, the relative infiltration abundance of the 24 immune cell types was further calculated by the ssGSEA algorithm. As shown in **Figure 8A**, the infiltration abundance of immune cells exhibited a high correlation with ALOX12 expression, especially effective memory T (Tem) cell, natural killer (NK) cell, and T helper 2 (Th2) cell. Furthermore, the hierarchical clustering method was employed to classify the samples into three subtypes (high, medium, and low immune infiltration). Consistently, a high expression of ALOX12 revealed a higher abundance of immune cell infiltration, and ALOX12 expression was significantly different between the high immune infiltrating subtype and the medium and low immune infiltrating subtypes (**Figures 8B, C**). As illustrated in **Figures 8D, E**, most of the 27 immune checkpoints were significantly different between the high and low groups, including *CD276*, *CD70*, *ICOS*, *CTLA4*, *PDCD1* (*PD-1*), *CD274* (*PD-L1*), *PDCD1LG2*, *BTLA*, *CD27*, *CD40*, *CD40LG*, *HHLA2*, *TNFRSF18*, *TNFRSF4*, *ICOSLG*, *TNFRSF9*, *TNFSF14*, *LAG3*, *HAVCR2*, *ENTPD1*, and *NCR3* (all *P* < 0.05), which provided potential ICB therapeutic targets for CRC patients. Previous studies indicated that patients with a high expression of PD-1 and PD-L1 benefited more from nivolumab and pembrolizumab (2). Of note, the expressions of *PD-1*, *PD-L1*, and *CTLA4* were all significantly upregulated in the ALOX12 highly expressed group (*P* < 0.0001), revealing the encouraging application value of immune checkpoint blockade (ICB) in CRC patients with a high *ALOX12* expression.

### High Expression of ALOX12 Suggested Higher ICB Clinical Benefit

TIDE indicated that the immunotherapy response rates of patients with high *ALOX12* expression were dramatically higher (*P* < 0.001; **Figure 8F**). In parallel, SubMap analysis performed that high group patients who responded to anti-CTLA-4 and anti-PD1 therapy exhibited high similarity (*P* < 0.05; **Figure S3A**). Both TIDE and Submap confirmed that high ALOX12 expression group patients benefit more from immunotherapy, especially anti-CTLA-4 and anti-PD1 treatments.

### CMap Analysis Determined Potential Compounds/Inhibitors for CRC

The candidate compounds of CRC that might target pathways related to ALOX12 were investigated. CMap, a systematic data-driven method to discover the relationships among chemicals, biological conditions, and genes, was further employed. As illustrated in **Figure S3B**, 15 potential CRC therapeutic agents that are highly associated with ALOX12 were finally observed.

## Discussion

CRC is a highly malignant cancer with dismal recurrence and mortality rates (34). Bevacizumab is an anti-angiogenic drug mainly applied in advanced mCRC. Past studies indicated that bevacizumab had obtained great benefits in combination with multiple treatment modalities such as chemotherapy, neoadjuvant chemotherapy, and immunotherapy (9). However, the response of CRC patients with different stages to bevacizumab was heterogeneous (35). Over the past years, immunotherapy has exhibited a huge sensation owing to its dramatic efficacy in the treatment of solid cancers. In 2017, ICI therapy was approved for the treatment of patients with advanced microsatellite instability (MSI-H) or DNA mismatch repair (dMMR) deficiency in CRC. Nonetheless, unlike dMMR/MSI-H CRC, immunotherapy performed limited benefits for other CRC patients (2). Considering the poor prognosis and the significant heterogeneity of bevacizumab response and immunotherapy in CRC, exploring a novel biomarker to comprehensively evaluate the prognostic and the response to bevacizumab and immunotherapy is of great significance.

To gain new insights into the mechanisms underlying bevacizumab response, we employed WGCNA to identify bevacizumab response-related modules. GO and KEGG enrichment analyses exhibited that most genes in these modules were enriched in cell proliferation and tumorigenesis-related pathways including DNA replication, P53 signaling pathway, cell cycle, and cell division pathways, indicating that the inhibition of cell proliferation processes might be a potential mechanism for bevacizumab to suppress the progression and migration of CRC. In the present study, patients with a higher ALOX12 expression exhibited a better bevacizumab response. Zhonghua Zheng et al. reported that the expression of ALOX12 facilitated the proliferation of tumor cells (36), which was proved in the colony formation, CCK-8 proliferation, and EdU incorporation assays, and consistently with the results of GO and KEGG.

Elegant studies have revealed that ALOX12 encodes arachidonic acid 12-lipoxygenase and is widely expressed in various cell types. Due to the function of regulating cell migration, platelet aggregation, and tumor cell proliferation, ALOX12 was mainly associated with the occurrence and procession of diseases like atherosclerosis, thrombosis, and tumors (36). In our research, a high expression of ALOX12 revealed worse OS, RFS, and PFS in CRC, which was consistent with previous reports in kidney, breast, and prostate tumors (37). GSEA enrichment analysis suggested that ALOX12 was tightly associated with tumor occurrence and metastasis pathways such as ECM receptor interaction, NOTCH signaling pathway, pathways in cancer, and positive regulation of GTPase activity. Additionally, based on the median expression of ALOX12, patients were divided into high and low groups. GSVA demonstrated that most of the tumor-related pathways exhibited significant differences between the high and low ALOX12 expression groups, further validating the important potential functions of ALOX12 in tumors.

Generally, similar links exist between landscape diversity and landscape function (38). In the present study, gene mutation frequencies, SNPs, and indels were not identical in the mutant landscape of ALOX12, suggesting that there were differences between the high- and low-expression groups in gene levels. Of note, the mutation frequency of RYR3 was significantly frequent in the high-expression group, indicating that RYR3 mutation drives the expression of ALOX12. Lina Zhang and Zhen-Hao Liu et al. reported that RYR3 mutations were closely related to the prognosis and metastasis of breast and combined hepatocellular cholangiocarcinoma (CHC) (39, 40). From what we can speculate, high ALOX12 expression CRC patients tend to exhibit a worse prognosis and were more likely to undergo metastasis; subsequently, it was verified in wound healing and transwell assays. In parallel, our results indicated that the deletion of chromosome fragment 17p12 inhibited the expression of ALOX12. Studies by Han et al. and Kim et al. demonstrated that the absence of 17p12 in multivarious cancers such as breast and serous ovarian cancer promoted the resistance to neoadjuvant chemotherapy (41, 42). Given this, we hypothesize that patients with low ALOX12 expression in CRC are more likely to develop chemotherapy resistance, while it needs further clinical and experimental verification.

As is known to all, TMB was regarded as a sign of immunotherapy response in some tumors. High TMB normally indicated a better immunotherapy response (43, 44). In our research, patients with a high ALOX12 expression displayed a higher TMB. Additionally, we investigated the relationship between ALOX12 expression and immune cells and checkpoints. The results suggested that the higher the expression of ALOX12, the more abundant the infiltration of immune cells. Moreover, differences in ALOX12 expression between the high immune infiltration subtype and the moderate and low immune infiltration subtypes were dramatically significant. Previous studies confirmed that CTLA-4, PD-1, and PD-L1 as immune checkpoints could prevent the immune system from killing cancer cells by inhibiting the autoimmunity (45, 46). As we expected, patients with a high expression of ALOX12 illustrated higher levels of PD-1, PD-L1, and CTLA-4, indicating that high ALOX12 expression patients benefit more from ICB therapy. On the basis of the above results, SubMap and TIDE methods were further employed to predict the benefits of ICB treatment for CRC patients. Consistently, patients with a high ALOX12 expression indicated better ICB benefits. Last but not least, we investigated the potential drug targets for CRC patients, and 15 potential CRC therapeutic agents associated with ALOX12 were finally observed, which provided new insights into precision therapy for CRC patients.

Although ALOX12 is a promising comprehensive biomarker, some limitations should be acknowledged. Firstly, although we comprehensively searched public databases for bevacizumab treatment cohorts, the sample size is still limited, and future studies should be conducted in a larger sample cohort. Secondly, all the samples in our study were retrospective; future validation of ALOX12 should be conducted in prospective fresh samples. Thirdly, due to limited CRC recurrence data, we only explored the predictive performance of ALOX12 for recurrence in TCGA cohort, and the results suggest that ALOX12 is a promising CRC recurrence-predictive marker; however, future validation using more recurrence cohorts is necessary.

In summary, based on systematic and comprehensive bioinformatics analyses and experimental verification, we identified a stable and powerful biomarker, which filled the gap in this field to comprehensively predict the bevacizumab response, prognosis (OS, RFS, and PFS), and immunotherapy effects for CRC patients. In addition, high expression in tumor tissues makes ALOX12 easier to be detected, increasing its utility for clinical applications. GSEA, GSVA, and multi-omics data analysis demonstrated that high ALOX12 expression patients were not only tightly related to tumor development and metastasis but also possessed potential benefits for chemotherapy. In conclusion, our study provided a powerful biomarker for CRC patients, which exhibited a dramatic significance in clinical therapeutic regimen optimization, prognostic risk assessment, precision treatment, and the individualized treatment regimen formulation of CRC.

## Data Availability Statement

The original contributions presented in the study are included in the article/**Supplementary Material**. Further inquiries can be directed to the corresponding authors.

## Ethics Statement

The studies involving human participants were reviewed and approved by the Ethics Committee Board of The First Affiliated Hospital of Zhengzhou University. The patients/participants provided their written informed consent to participate in this study.

## Author Contributions

ZL and SW contributed to the study design and data analysis. HX contributed to the manuscript writing. XH and JR contributed to the project oversight and manuscript revisiting. SW and XG collected the samples and generated the data. YR, QD, LL, JZ, and PL contributed to the manuscript revisiting. All authors contributed to the article and approved the submitted version.

## Conflict of Interest

The authors declare that the research was conducted in the absence of any commercial or financial relationships that could be construed as a potential conflict of interest.

## Publisher’s Note

All claims expressed in this article are solely those of the authors and do not necessarily represent those of their affiliated organizations, or those of the publisher, the editors and the reviewers. Any product that may be evaluated in this article, or claim that may be made by its manufacturer, is not guaranteed or endorsed by the publisher.

## Supplementary Material

Supplementary Figure 1 Immunohistochemical results of tumor and normal CRC tissues.

Supplementary Figure 2 Topological overlap matrix and the prognostic analysis of ALOX12. **(A)** Topological overlap matrix of selected genes. **(B)** Kaplan-Meier survival analysis of ALOX12 in our in-house cohort. **(C–G)** Univariate Cox regression analysis of six genes with AUC>0.7 in TCGA-CRC, GSE72970, and our cohort.

Supplementary Figure 3 The result of Submap algorithm **(A)** and Connectivity map analysis **(B)**.
