# Supplementary figures and images for: ALOX12: A Novel Insight in Bevacizumab Response, Immunotherapy Effect, and Prognosis of Colorectal Cancer
Source: Front Immunol. 2022 Jun 27;13:910582. doi: 10.3389/fimmu.2022.910582 (PMC9271859; doi:10.3389/fimmu.2022.910582)

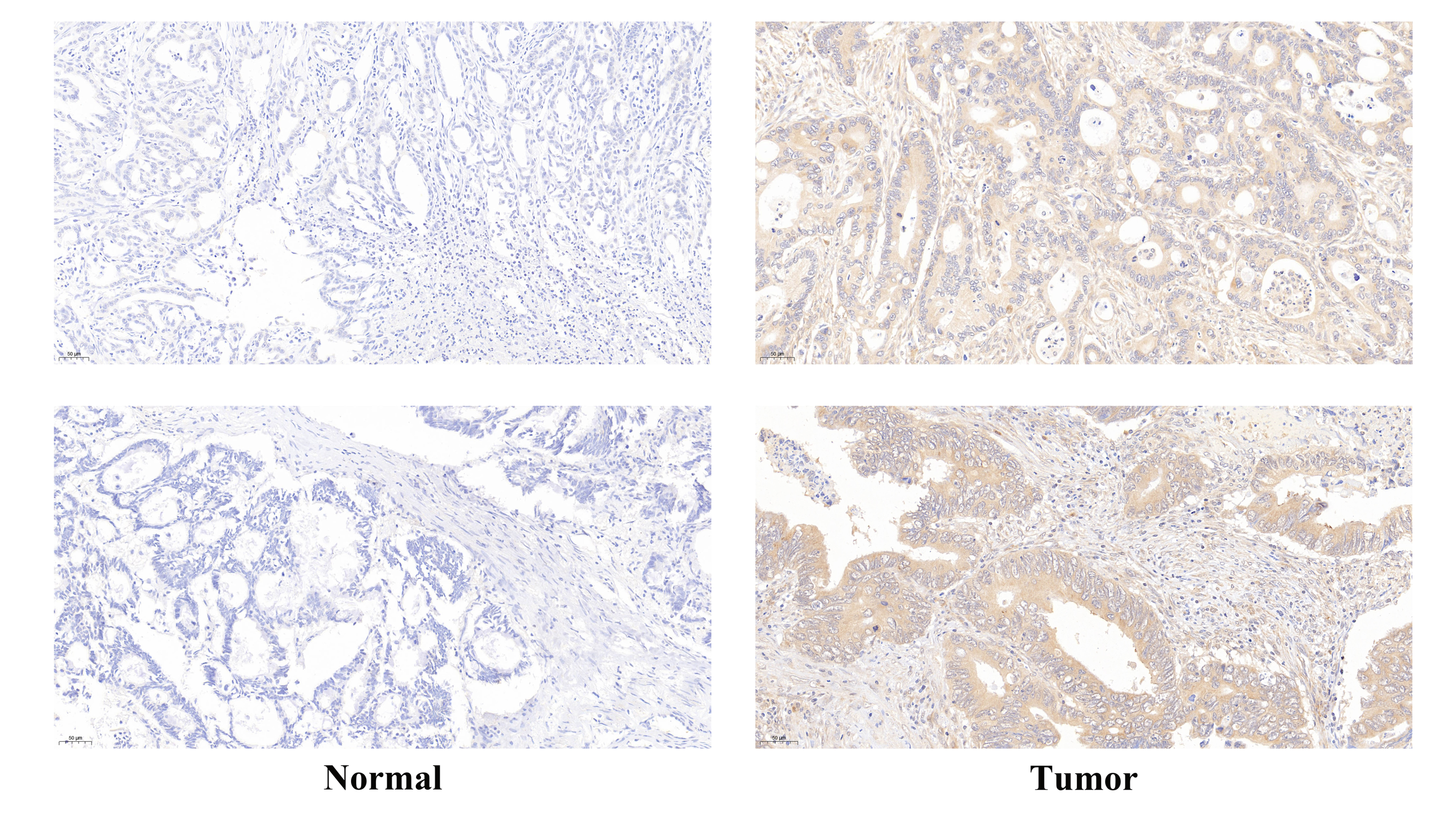

Supplement: Supplementary Figure 1 — Immunohistochemical results of tumor and normal CRC tissues. [file Image_1.jpeg]

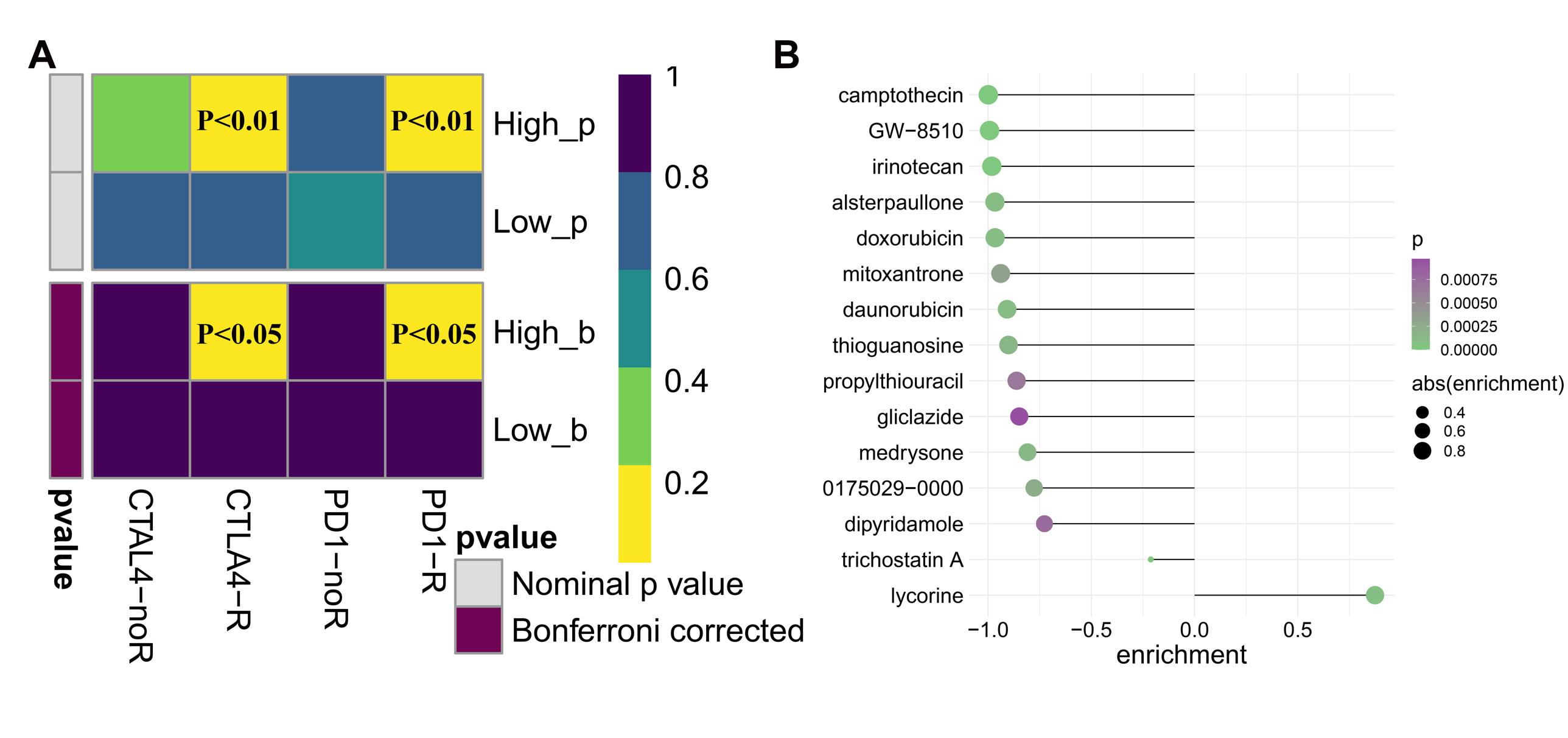

Supplement: Supplementary Figure 3 — The result of Submap algorithm (A) and Connectivity map analysis (B). [file Image_3.jpeg]
